# MLLT11-TRIL complex promotes the progression of endometrial cancer through PI3K/AKT/mTOR signaling pathway

**DOI:** 10.1080/15384047.2022.2046450

**Published:** 2022-03-06

**Authors:** Jingnan Liao, Huan Chen, Mingming Qi, Jinjin Wang, Mingyuan Wang

**Affiliations:** a National Clinical Research Center for Geriatric Disorders, Xiangya Hospital, Central South University, Changsha, Hunan, China; b Institute of Reproductive and Stem Cell Engineering, School of Basic Medical Science, Central South University, Changsha, Hunan, China; c Department of Gynaecology, The Affiliated Zhuzhou Hospital Xiangya Medical College, Central South University, Zhuzhou, Hunan, China; d Department of Obstetrics and Gynecology, The Second Xiangya Hospital of Central South University, Changsha, Hunan, China; e Department of Geratic Surgery, Xiangya Hospital, Central South University, Changsha, Hunan, China

**Keywords:** Endometrial cancer (EC), MLLT11, TRIL, AKT, PI3K/AKT/mTOR signaling pathway

## Abstract

Endometrial cancer (EC) is a gynecological malignant tumor characterized by high incidence. EC occurrence and development are regulated by numerous molecules and signal pathways. There is a need to explore key regulatory molecules to identify potential therapeutic targets to reduce the incidence of EC. Treatment by targeting a single molecule is characterized by poor efficacy owing to the development of resistance and significant side effects. The current study explored potential candidates in EC by integrating bioinformatics analysis and in vivo and in vitro experimental validation to circumvent the limitation of low efficacy of currently used molecules. Molecular dynamics simulations provide details at the molecular level of intermolecular regulation. In the current study, MLLT11 and TRIL were identified as important regulatory molecules in EC. The two molecules formed a heteromultimer by binding to AKT protein, which induced its phosphorylation of threonine at position 308. Ultimately, the complex stimulates PI3K/AKT/mTOR signaling pathway, a pivotal pathway in tumors. The findings of the current study show a novel complex, MLLT11-TRIL, which can act as AKT protein agonist, thus inducing activity of PI3K/AKT/mTOR signaling pathway. Targeting MLLT11 and TRIL simultaneously, or blocking the formation of the MLLT11-TRIL complex, can abrogate progression of EC.

## 1. Introduction

Endometrial cancer (EC) is a common gynecological malignant tumor and the fifth most common cause of cancer deaths in the United States.^1^^, 2^ It exhibits one of the most extreme tumor heterogeneity among human cancers.^3^ With the rapid development of molecular biology tools, several molecular signal transduction pathways have been shown to mediate the occurrence and development of EC. Thus, key pathway molecules present potential therapeutic targets.^4^ Since interventions targeting a single molecule have been associated with poor efficacy and side effects,^5^ most studies are evaluating molecular mechanisms and development of therapies against multiple targets in EC.

According to the pathogenesis of the disease, it can be divided into two types. Type I is estrogen dependent, accounting for the majority of EC. Type II is estrogen-independent and has a worse prognosis than type I.^6^ Among them, type I EC encodes genetic changes that are mostly associated with PI3K and Wnt pathway signaling abnormalities, KRAS mutations, and PTEN inactivation.^4^ In addition, PI3K/AKT/mTOR, the most important signal pathway, plays a pivotal role in tumor activities.^7^

AKT (Protein kinase B, PKB) is a serine/threonine kinase, which regulates many biological processes, such as metabolism, proliferation, cell survival, growth, and angiogenesis.^8^ It is a downstream signal for phosphatidylinositide 3-kinases (PI3K). Besides, many of its activators such as receptor tyrosine kinases, integrins, B cell and T cell receptors, cytokine receptors, and G protein-coupled receptors have been reported.^9^ In addition, 3-phosphoinositide-dependent protein kinase-1 (PDK1) has been shown to phosphorylate AKT at threonine 308, leading to its partial activation.^10–12^ The phosphorylation of threonine 308 could widely affect the downstream regulation of AKT, thus modulating the activity of PI3K/AKT/mTOR signaling pathway. It is, therefore, feasible to speculate that the phosphorylation of AKT protein might mediate tumorigenesis in EC.

MLLT11, also known as af1q, is located on human chromosome 1 and encodes a 9 kDa protein. MLLT11 was originally found in leukemia patients as an oncogene.^13^ Although the biological function of MLLT11 is largely unknown, the potential cancer-promoting effect of the gene has been proposed in breast cancer, colorectal cancer, and osteosarcoma.^14–16^

TRIL is expressed in many tissues including brain, spinal cord, lung, kidney and ovary, which may be related to the functional role of TLR4 in the brain.^17^ However, so far, the role of TRIL in tumor development has not been reported.

Here, we showed that MLLT11-TRIL complex acts as an AKT agonist and stimulates the activity of the PI3K/AKT/mTOR signaling pathway. Thus, dual targeting of MLLT11 and TRIL might help prevent the progression of EC. Coupled with our previous findings on protein complex,^18^ we demonstrate that binding of MLLT11-TRIL has catalytic effect on phosphorylation of AKT-threonine 308, and might be a therapeutic target in EC.

## 2. Materials and methods

### 2.1 Transcriptome data acquisition and pre-processing

Transcriptome microarray data for 37 endometrial tissues and their corresponding clinical information were obtained from the Metabolic gEne RApid Visualizer (MERAV, http://merav.wi.mit.edu) database.^19^ To control for the potential bias caused by a single data set, we collected transcriptome level 3 data of uterine corpus endometrial carcinoma (UCEC) and corresponding clinical prognostic information from the Cancer Genome Atlas Project (TCGA, https://www.cancer.gov/tcga) database.^20^ We conducted analysis of the obtained data against a standardized reference.

### 2.2 Bioinformatics analysis

We established a weighted gene co-expression network analysis (WGCNA) of the 37 endometrial tissue data in MERAV, following our previously described protocol.^21^ Based on the characteristics of the data, we characterized the phenotypes according to clinical stage, pathological grade, and their cancerous status. Since there were correlations between the different modules and phenotypes, we extracted the modules with the strongest correlation with the phenotypes. Besides, we conducted signal pathway enrichment analysis using g:Profiler (https://biit.cs.ut.ee/gprofiler/gconvert.cgi).^22^

To screen out the pivotal gene pairs, we profiled the gene expression correlation in candidate modules using the Pearson correlation coefficient (r) at a significance level of p < .05. In addition, we analyzed and extracted genes that had significant effect on the prognosis of UCEC in the TCGA dataset. Similarly, we established an expression correlation network to sieve important gene pairs. Gene pairs shared by the two sets of data were selected as candidate genes for subsequent studies.

### 2.3 Cell lines and collection of clinical specimen

Immortalized human endometrium cell lines, CRL-4003 and T-HESC were purchased from the Shanghai Institute for Biological Science, Chinese Academy of Sciences (Shanghai, China). On the other hand, human endometrial carcinoma lines, AN3, ECC-1, RL95-2, KLE, Ishikawa and Hec1A lines were obtained from our laboratory.

In addition, we collected endometrial tissues from 50 patients who had been diagnosed with EC at the Zhuzhou Central Hospital, Xiangya Medical College, Central South University from January 1, 2020 to January 1, 2021. The EC was pathologically determined and the patients gave informed and signed consent (clinicopathologic characteristics were shown in the supplementary Table S3). The study was approved by the Ethics Committee of Zhuzhou Central Hospital, Central South University (Zhuzhou, China).

### 2.4 Reagents and antibodies

We purchased multiple PI3K/AKT signaling inhibitors and activators from Selleck (Selleck Chemicals, Shanghai, China). They include LY294002 (Cata No. S1105), a small molecule inhibitor of PI3K; MK-2206 (Cata No. S1078), a highly selective AKT inhibitor; Rapamycin (Cata No. S1039), a specific mTOR inhibitor and a cell permeable PI3K phosphopeptides activator 740 Y-P (Cata No. S7865). R3 IGF-1 (Cata No. I1146), a recombinant analog of IGF-1, was purchased from Sigma-Aldrich (St. Louis, MO, USA). DMSO was used as a vehicle for all the negative control assays. All reagents were used at the manufacturers’ recommended concentration.

We used PI3K-p110δ (ab109006, Abcam), PTEN (ab267787, Abcam), p-AKTThr308 (ab38449, Abcam), pan-AKT (ab18785, Abcam), p-mTORSer2448 (ab109268, Abcam), mTOR (ab134903, Abcam), GAPDH (ab8245, Abcam), MLLT11 (PA5-81879, Invitrogen), TRIL (PA5-23454, Invitrogen) and IgG antibody (Cat#2729, Cell Signaling) as primary antibodies. Goat anti-rabbit IgG (ab150077, Abcam) was used as the secondary antibody.

### 2.5 Cell culture and transfection

The cells in logarithmic growth phase were seeded in 6 wells (1 × 10^5^ cells per well) and cultured overnight in a 5% CO_2_ incubator at 37°C. At 65%˜85% confluence, lipofectamine 3000 reagent was used for the transfection of siRNA control (si-Con), MLLT11 siRNA (si-MLLT11) and TRIL siRNA (si-TRIL) into the cells. After 48 hours, we analyzed the transfection efficiency using qRT-PCR and Western blot assays.

### 2.6 Quantitative real-time PCR (qRT-PCR)

Total RNA was extracted from the cells in the logarithmic growth phase using Trizol. The quality of the RNA was evaluated by ultraviolet spectrophotometry, and then cDNA was synthesized using reverse transcription kit (Takara, Japan). The expression of MLLT11 and TRIL mRNA in the cells was evaluated by qRT-PCR using the following primers: MLLT11, forward 5’-GGACCCTGTGAGTAGCCAGTA-3’ and reverse 5’-CAGCTCCGACAGATCCAGT-3’; TRIL, forward 5’-GTACCTGGGGAACAACCTCTT-3’ and reverse 5’-GCAGCTTGACTAGACTCTCCA-3’; GAPDH, forward 5’-CCAGCAAGAGCACAAGAGGAAGAG-3’ and reverse 5’-GGTCTACATGGCAACTGTGAGGAG-3’. GAPDH as internal reference. The relative expression of the MLLT11 and TRIL mRNA was calculated by 2-ΔΔCT method.

### 2.7 MTT and wound scratch healing assay

Cell proliferation was detected by 3-(4,5-dimethyl-2-thiazyl)-2, 5-diphenyl-2 H-tetrazolium bromide (MTT) assay and was measured by optical density D (λ) at a wavelength of 490 nm using a BioTek Gen5 system (BioTek, USA).

Cell migration was assessed using a wound scratch healing assay using the previously described protocol.^23,24^ First, monolayer cells were prepared. The cell density was 5 ~ 10 × 10^5^ cells/ml were placed in 24 well plates (500 UL per well), and 10% fetal bovine serum was added to RMPI 1640 medium, cultured for 16 ~ 24 h to form monolayer cells. The cells were grouped according to different needs. Scratched the monolayer cells with a 10 UL pipette head, washed it with PBS 3 times, and replaced it with RMPI 1640 culture medium. Sucked the culture medium, washed it with PBS 3 times, observed, and took photos under an inverted fluorescence microscope.

### 2.8 Protein preparation and Western blot

Cells were harvested, and then total protein lysate was prepared using a prechilled RIPA buffer with proteinase and phosphatase inhibitor. The protein concentration was determined using BCA assay (Sigma-Aldrich, USA) for the separation of nuclear and cytoplasmic proteins in cells. We extracted nuclear and cytoplasmic proteins from the cells according to the instructions of the nucleocytoplasmic separation kit. Briefly, cytoplasmic protein extraction reagents A and B were added to the collected cell pellet successively, the system was mixed evenly, and the supernatant liquid obtained by ultracentrifugation was the cell cytoplasmic protein. Nuclear protein extraction reagent was added to the precipitate, and the supernatant liquid obtained after ultracentrifugation was nuclear protein. We used Western blotting to detect the subcellular localization of the protein of interest.

The protein samples were resolved in sodium dodecyl sulfate-polyacrylamide gel electrophoresis (SDS-PAGE), then transferred onto polyvinylidene difluoride (PVDF) membranes. The blots were blocked in skimmed milk for 2 h and then incubated with primary antibodies overnight at 4°C. The blots were washed in PBS before incubation with secondary antibody. The optical density of the target protein band was analyzed by the Bio-Rad imager.

### 2.9 Immunohistochemistry (IHC)

For IHC, tissue sections were dewaxed, hydrated, and then placed in PBS buffer. The sections were soaked in hydrogen peroxide at room temperature and then incubated with primary antibody overnight at 4°C. The sections were then incubated with secondary antibody and restained with hematoxylin. Thereafter, the slices were dehydrated with ethanol at different concentration gradients and then sealed with neutral gum. We then examined the tissue morphology and photographed under a microscope. Add up all the optical density values of each Brown point on the picture and divide the obtained value by the area of the effective target distribution area. Use ImageJ software to detect the percentage of positive cells and total cells in one field of vision, and then take 10 same fields to calculate the average index.

### 2.10 Cell apoptosis assay

The cells at a concentration of 1 × 10^5^ cells/well were inoculated into 6-well plates and then digested with trypsin. After washing in PBS, 10 μL Annexin V-FITC was added and incubated at room temperature for 15 min in darkness. The cells were stained with 5 μL PI solutions, and then they were evenly mixed and incubated for 5 min in darkness. The percentage apoptosis was analyzed by flow cytometry.

### 2.11 Co-immunoprecipitation (Co-IP)

We extracted proteins from each group of cells and then used Protein A + G Agarose to remove nonspecific binding. We incubated the extracted protein samples with primary antibody overnight and then resolved them in an SDS-PAGE, before performing Western blot analysis.

### 2.12 Animal studies and lentivirus infection

Female 4-week-old athymic BALB/C nude mice were randomly divided into groups. The cultured cells were digested with trypsin and resuspended in PBS buffer. 100 μL of 2 × 10^6^/100 μL cell suspension was subcutaneously injected into the left armpit of each nude mouse. The tumor volume was measured after every 3 days. On the 30th day, the mice were sacrificed and then tumors were removed.

For gene therapy, five mice in each group were injected with lentivirus. We performed multi-point injection, once every three days. The treatment groups included: MLLT11 shRNA lentivirus 0.1 ml/(5 × 10^8^ PFU/ml); TRIL shRNA lentivirus 0.1 ml/(5 × 10^8^ PFU/ml); and MLLT11 shRNA 0.05 ml/(5 × 10^8^ PFU/ml) or TRIL shRNA 0.05 ml/(5 × 10^8^ PFU/ml) while the negative control group was used scramble shRNA lentivirus 0.1 ml/(5 × 10^8^ PFU/ml). The treatments were given on the 15th day after tumor inoculation. All the lentiviruses were constructed by Genechem (Shanghai, China). The animal study was approved by the Animal Ethics Committee of Central South University (Changsha, China).

### 2.13 Protein modeling and molecular docking

The MLLT11 protein model was constructed by Robetta Server (http://robetta.bakerlab.org)^25^ while TRIL or AKT1 proteins were modeled by “Modeler 9.20” software using 2id5 and 3o96, respectively, as PDB templates (supplementary Table S1).

Protein docking is a protein–protein interaction docking program based on fast Fourier transform, which is provided and maintained by Zhiping Weng Laboratory of Massachusetts Medical School. The program is mainly used to search for all possible binding patterns in the space obtained by translation and rotation between proteins, and to evaluate each binding model using an energy-based scoring function.

### 2.14 Molecular dynamics simulations

Molecular dynamic (MD) simulations were performed with GROMACS 2018.4 software. The protein complex system was placed at the center of a cube box with a side length of 12 nm, and the distance between each atom of the protein and the box was greater than 1.0 nm. We then randomly filled the box with water molecules. A 1000-step energy minimization simulation was used to optimize the system. In this study, we used the Verlet leapfrog algorithm to solve Newton’s equation of motion while the Lennard Jones function was used to calculate the van der Waals force. In addition, the LinCS algorithm was used to constrain the bond length of all atoms while Particle mesh ewald (PME) was used to calculate the long-range electrostatic interaction. The MD simulations were carried out under isothermal isobaric ensemble. The time of MD simulation for the wild type (WT; no mutation) and phosphorylated mut (AKT1 protein Thr308 phosphorylation) was 50 ns.

### 2.15 Statistical analysis

Data were shown as a mean ± standard deviation (x± SD). The difference between the two groups was analyzed by a t-test, while the Pearson’s correlation coefficient was used for the correlation analyses. In addition, survival analysis was performed using Kaplan–Meier survival curves. * p < .05, ** p < .01 and *** p < .001 were considered statistically significant differences. Statistical analyses were performed on R software and GraphPad Prism software (GraphPad Software, USA).

## 3. Results

### 3.1 In silico screening of valuable genes

**Figure 1.**
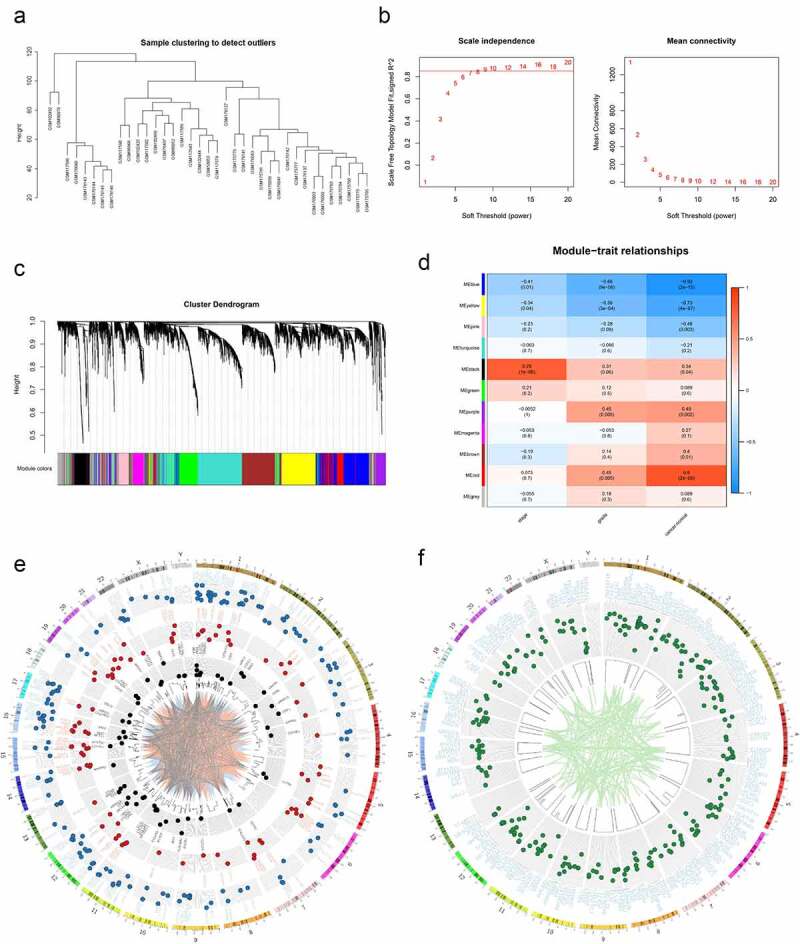
Preliminary screening of valuable genes. (a) Cluster dendrogram of EC samples in MERAV. (b) Left: Analysis of the scale-free fit index for various soft-thresholding powers (β). Right: Analysis of the mean connectivity for various soft-thresholding powers (β). (c) Dendrogram of genes cluster. Each module was distinguished by different colors. (d) Heat map of the correlation between modules and phenotypic traits of EC. (e) Circos plot of the genes in black, red and blue modules and their co-expression network. (f) Circos plot of the genes with significant differences in survival analysis in TCGA.

For the WGCNA, we performed cluster tree analysis on all the samples and showed no presence of obvious outliers (Figure 1(a)). The analysis showed that a soft threshold β = 9 (R2 > 0.85) yields the lowest adjacency coefficient, which was the best soft threshold for scale-free network (Figure 1(b)). Genes were clustered into 11 modules with each module distinguished by different colors (Figure 1(c)). Our analysis on the correlation of each module with clinical stage, pathological grade or status of EC. We showed that the black module had the highest correlation with EC clinical stage (rp = .78, P = 1 × 10^−8^, Figure 1(d)). On the other hand, the red and blue modules had the strongest correlation with cancer (positive and negative correlations; Red: rp = .8, P = 2 × 10^−9^; Blue: rp = −0.92, P = 2 × 10^−15^, Figure 1(d)). We further showed the location of genes on chromosome as well as the network of significant expression correlation between two genes (P < .05, Figure 1(e)). To narrow the screening, the transcriptome EC data from the TCGA data were incorporated and then survival analyses were performed. We then extracted genes with significant differences (P < .05) in the survival analysis. In addition, we defined positions of individual genes on chromosomes and then generated a network with significant correlation between genes (P < .05, Figure 1(f)).

### 3.2 MLLT11-TRIL with PI3K/AKT/mTOR signaling pathway defines the development of EC

**Figure 2.**
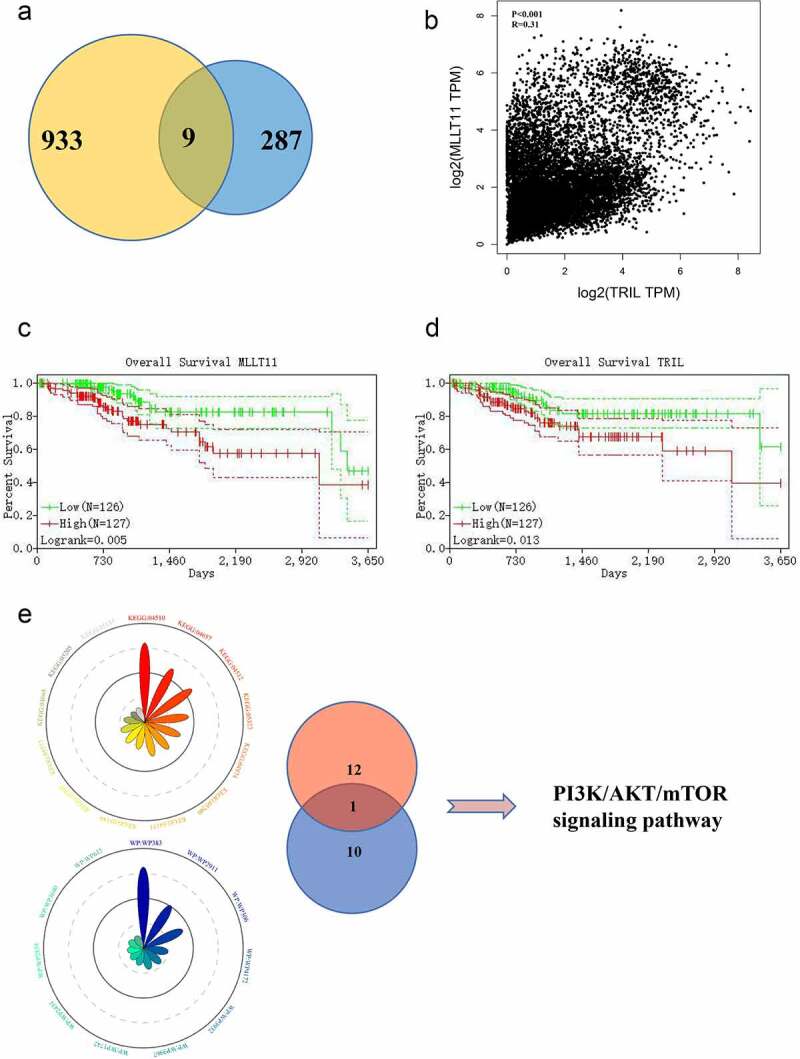
Bioinformatics analysis targets MLLT11-TRIL and PI3K/AKT/mTOR signaling pathway. (a)The Venn diagram of the gene pairs involved in Figure 1e and F. (b) Scatter plot of MLLT11 and TRIL expression in TCGA database. (c) Overall survival of MLLT11 based on Kaplan–Meier plotter with a 95% confidence interval. (d) Overall survival of TRIL based on Kaplan–Meier plotter with a 95% confidence interval. (e) The genes in the black, red and blue modules were enriched in two aspects: “KEGG” and “WikiPathways”, and displayed on the Radar charts.

Our gene pair analysis showed a total of 9 pairs of genes in the intersection (Figure 2(a)). We then selected MLLT11-TRIL as the candidate regulatory factor for subsequent studies (Figure 2(b)). This is because the survival analyses demonstrated that the higher expression of MLLT11 and TRIL was correlated with poorer disease prognosis (Figure 2(c,d)).

The genes in red, blue, and black modules were enriched in KEGG and Wikipathways. The data showed significant enrichment of the PI3K/AKT signaling pathway (Figure 2(e)). Thus, the PI3K/AKT signaling pathway might be a key molecular pathway in the pathogenesis of EC.

### 3.3 Validated in vivo, in vitro and in clinical tissues

**Figure 3.**
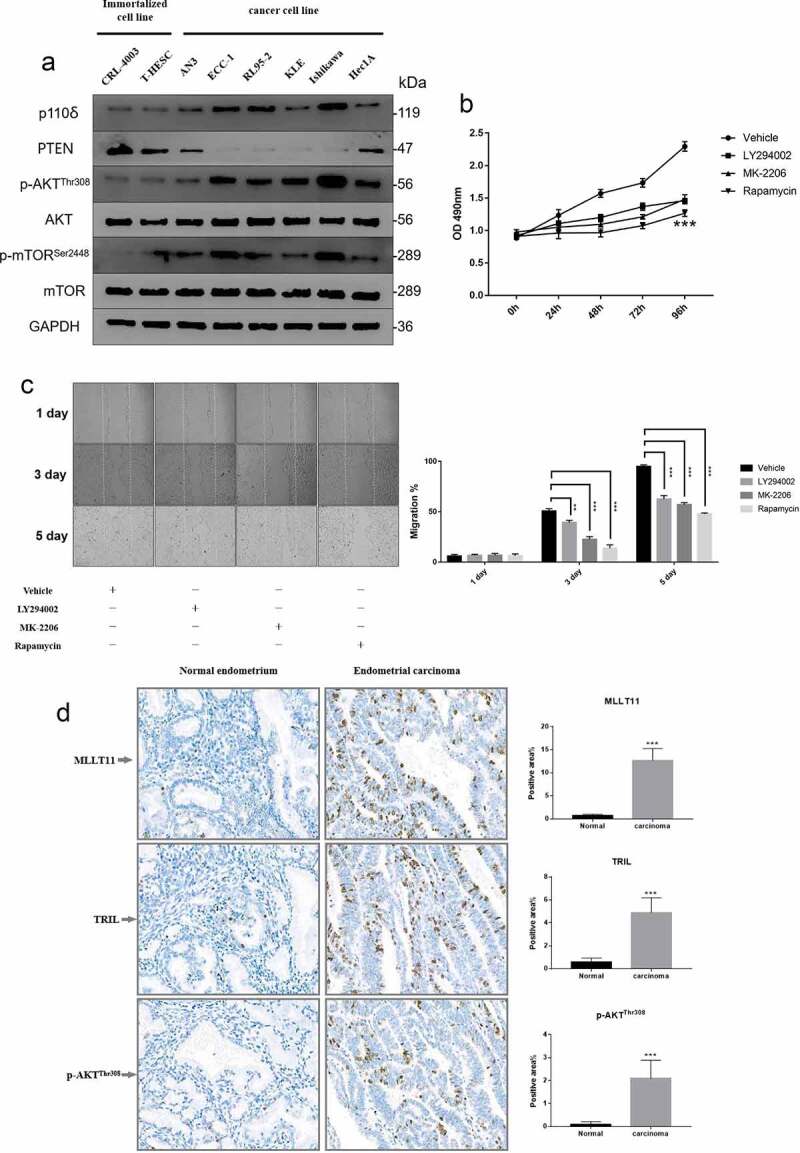
Cell line screening and tissue validation. Western blot was used to detect the expression levels of representative molecules in the PI3K/AKT/mTOR signaling pathway in 8 endometrial cell lines from different sources. (b) MTT assay was used to detect the cell viability of Ishikawa cells at different time points after LY294002, MK-2206 and Rapamycin treatment. *** p < .001. (c) The wound scratch healing assay detected the migration ability of Ishikawa cells at different time points after LY294002, MK-2206 and Rapamycin treatment. ** p < .01 and *** p < .001. (d) The expressions of MLLT11, TRIL and p-AKT^Thr308^ in normal endometrium and endometrial carcinoma were compared by IHC. *** p < .001.

We tested the expression of representative proteins in the PI3K/AKT/mTOR signaling pathway in eight endometrial cell lines. Three of the cell lines were normal endometrial cells while five cell lines were EC cells of different origins. The results showed upregulation of p110δ (a subunit of PI3K), p-AKT/AKT and p-mTOR/mTOR in EC cell lines compared to those in normal endometrial cell lines. On the other hand, the expression of PTEN was suppressed in EC cell lines (Figure 3(a)). These data demonstrated that the overall activity of the PI3K/AKT/mTOR signaling pathway in the EC cells was higher compared to the normal endometrial cells.

We then selected the EC cell line, Ishikawa, and used chemical inhibitors to evaluate the biological function of PI3K/Akt/mTOR signaling pathway. Cell viability and wound scratch healing assays demonstrated that LY294002, MK-2206, and Rapamycin could significantly inhibit the proliferation and migration of the Ishikawa cells, respectively (Figure 3(b,c)). These results indicated that the activation of PI3K/AKT/mTOR signaling pathway could significantly promote the proliferation and migration of Ishikawa cells.

Furthermore, we used immunohistochemistry to assess the expression of the tissue specimens from EC patients. We showed that MLLT11, TRIL, and p-AKT^Thr308^ were significantly expressed in tumor tissues compared with the normal endometrial tissues (Figure 3(d)).

**Figure 4.**
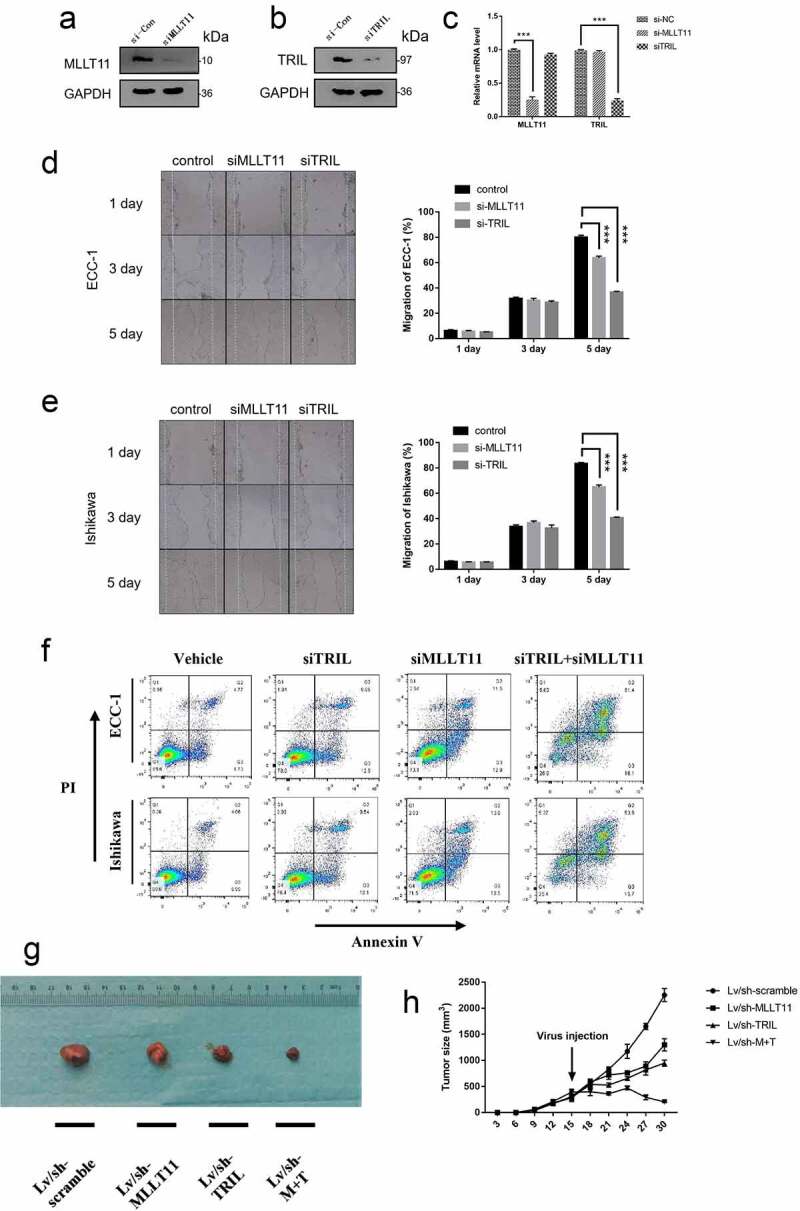
Effects of MLLT11 and TRIL on tumor phenotype. Western blot was performed to verify the effect of siRNA inhibition on the intracellular expression of (a) MLLT11 and (b) TRIL. (c) Suppression of MLLT11 and TRIL at the RNA level by siRNA was determined by qPCR. *** p < .001. Wound scratch healing assay was performed to detect the alteration of migration ability of (d) ECC-1 and (e) Ishikawa cells after MLLT11 and TRIL inhibition by siRNA. *** p < .001. (f) Flow cytometry was used to detect the effect of apoptosis rate after inhibition of MLLT11, TRIL, and combined inhibition of MLLT11 and TRIL by siRNA, respectively, in ECC-1 and Ishikawa cells. (g) The photo of nude mice tumor tissues in the lentivirus knockdown MLLT11, TRIL and MLLT11-TRIL co-knockdown groups (Lv/sh-MLLT11, Lv/sh-TRIL and Lv/sh-M + T) and its control group (Lv/sh-scramble). (h) Line graphs of tumor size of nude mice in the MLLT11, TRIL, MLLT11-TRIL co-knockdown and Lv/sh-scramble groups at different time points.

To further evaluate the biological functions of MLLT11 and TRIL, we used two independent siRNAs to suppress their expression in the cell, respectively. The data showed that si-MLLT11 and si-TRIL inhibited the expression of MLLT11 and TRIL proteins in the Ishikawa cell lines, respectively (Figure 4(a,b)). In addition, the qPCR results showed that si-MLLT11 and si-TRIL could, respectively, inhibit their mRNA expression levels. Moreover, si-MLLT11 did not show obvious effect on the TRIL mRNA level, and vice versa (Figure 4(c)). These illustrated the specificity of the si-RNA.

We selected two types of cells, ECC-1 and Ishikawa, and performed a wound scratch healing assay. The data showed that si-MLLT11 and si-TRIL could inhibit the migration of the two cells (Figure 4(d,e)). In addition, apoptosis assay showed that si-MLLT11 and si-TRIL could enhance the apoptosis of the ECC-1 and Ishikawa cells. Suppression of the expression of both MLLT11 and TRIL led to a significant increase in the apoptosis rate of the two cells (Figure 4(f), supplementary Figure S1).

We then performed tumorigenesis assays in nude mouse. In the first 15 days before gene intervention, there was no significant difference in tumor growth among the mice in the study groups. After administration of the lentivirus into each group for genetic manipulation, the data showed that suppression of MLLT11 and TRIL expression inhibited tumor growth relative to the wild-type control group (LV/sh-MLLT11 and LV/sh-TRIL, Figure 4(g,h)). As expected, simultaneous knockdown of both MLLT11 and TRIL led to significant inhibition of tumor growth compared with either the control group or the single knockdown group (LV/sh-M + T, *** p < .001, Figure 4(g,h)). These data demonstrated that that MLLT11-TRIL plays an important role in the growth of EC.

### 3.4 Interaction between the MLLT11-TRIL and PI3K/AKT/mTOR signaling pathway

**Figure 5.**
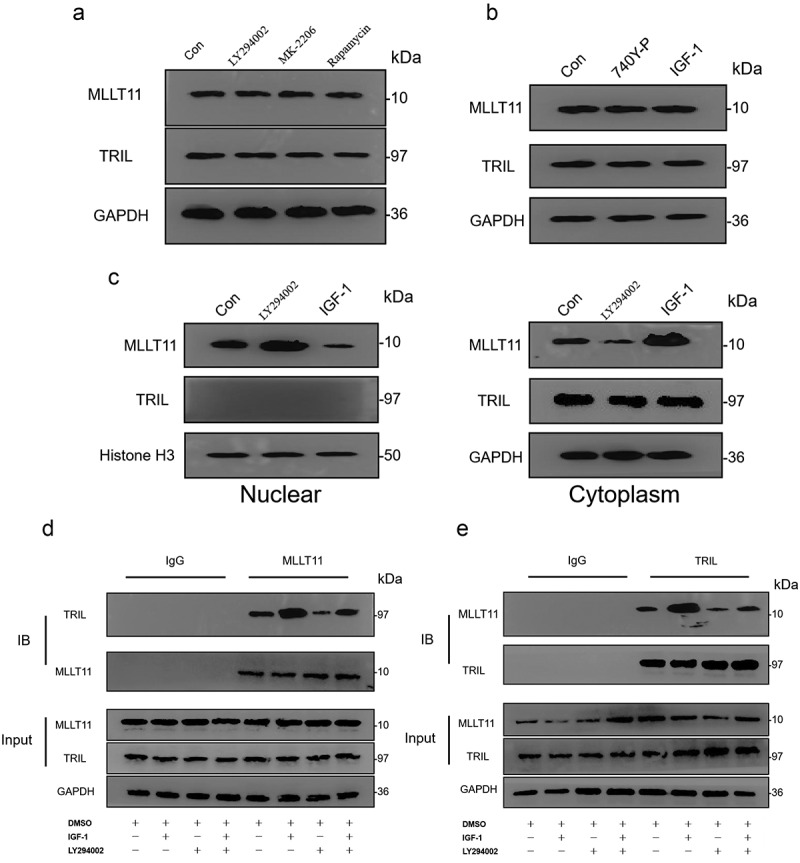
Interaction between the MLLT11-TRIL and PI3K/AKT/mTOR signaling pathway. Western blot was used to detect the effects of LY294002, MK-2206 and Rapamycin on the protein expression of MLLT11 and TRIL in cells. (b) Western blot was used to detect the effects of 740Y-P and IGF-1 on the protein expression of MLLT11 and TRIL in cells. (c) Isolation of nuclear and cytoplasmic proteins to examine the effects of LY294002 and IGF-1 on the subcellular distribution of MLLT11 and TRIL. Co-IP detected the interaction between cell endogenous protein MLLT11 and TRIL. The immunoprecipitates were immunoblotted (IB) with TRIL (d) or MLLT11 (e) antibody.

Next, we explored whether the activity of the PI3K/AKT/mTOR signaling pathway could affect the expression of both MLLT11 and TRIL. We applied inhibitors and activators of the PI3K/AKT/mTOR signaling pathway in Ishikawa cells. Western blot analysis showed that LY294002, MK-2206, and Rapamycin had no effect on the total expression of MLLT11 and TRIL protein (Figure 5(a)). Similarly, the agonists 740Y-P and IGF-1 did not change the total protein expression of both MLLT11 and TRIL (Figure 5(b)). We detected nuclear and cytoplasmic proteins separately and found that MLLT11 tended to be expressed in the nucleus after LY294002 treatment, while MLLT11 protein tended to be expressed in the cytoplasm after IGF-1 stimulation. The expression of TRIL protein was mainly in the cytoplasm, and LY294002 and IGF-1 could not change its subcellular distribution (Figure 5(c)). These findings indicated that the activation of the PI3K/AKT/mTOR signaling pathway might lead to changes in the subcellular distribution of the MLLT11 proteins.

Co-IP assays demonstrated that MLLT11 and TRIL interacted with each other in the cell. Stimulation of the cells by IGF-1 led to increased binding of the two proteins. As expected, application of the LY294002 inhibitor inhibited the interaction of the two proteins (Figure 5(d,e)). Thus, the interaction between MLLT11 and TRIL in cells was modulated by the activity of PI3K/AKT/mTOR signaling pathway. Together with the previous results, we speculated that the PI3K/AKT/mTOR signaling pathway might be affecting the binding of MLLT11 and TRIL but not the expression of the proteins.

### 3.5 MLLT11-TRIL binds to AKT and promotes its phosphorylation

**Figure 6.**
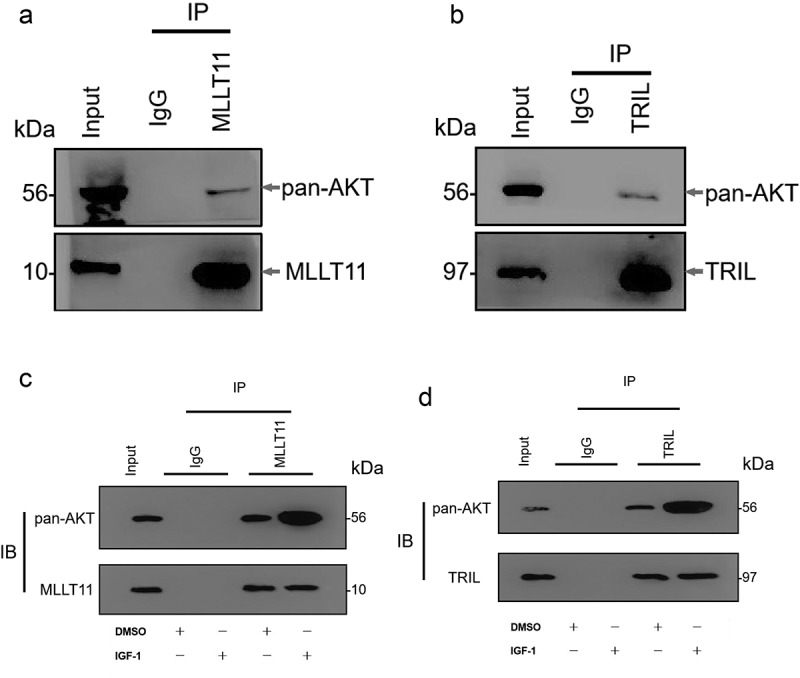
Co-IP detected the interaction between MLLT11-TRIL and AKT. The cell lysate was immunoprecipitated with IgG, MLLT11 (a) or TRIL (b) antibody. And immunoprecipitates were immunoblotted (IB) with pan-AKT antibody. After IGF-1 treated the cells, the above experiment was repeated. The immunoprecipitates were immunoblotted with pan-AKT antibody. The control group was treated with DMSO.

Our further analyses showed that the TRIL protein and AKT proteins bind to each other in Ishikawa cells. At the same time, MLLT11 protein was shown to bind and interact with AKT protein (Figure 6(a,b)). Whereas there was weak binding with the total AKT, use of IGF-1 to activate the PI3K/AKT/mTOR signaling pathway significantly enhanced the interaction between TRIL protein and AKT protein (Figure 6(c)). Similarly, there was significant enhancement of the interaction between MLLT11 protein and AKT protein (Figure 6(d)). Therefore, we speculated that the activation of PI3K/AKT/mTOR signaling pathway can promote the binding of MLLT11-TRIL complex to AKT protein.

### 3.6 In silico *analysis of the MLLT11/TRIL/p-AKT complex formation*

**Figure 7.**
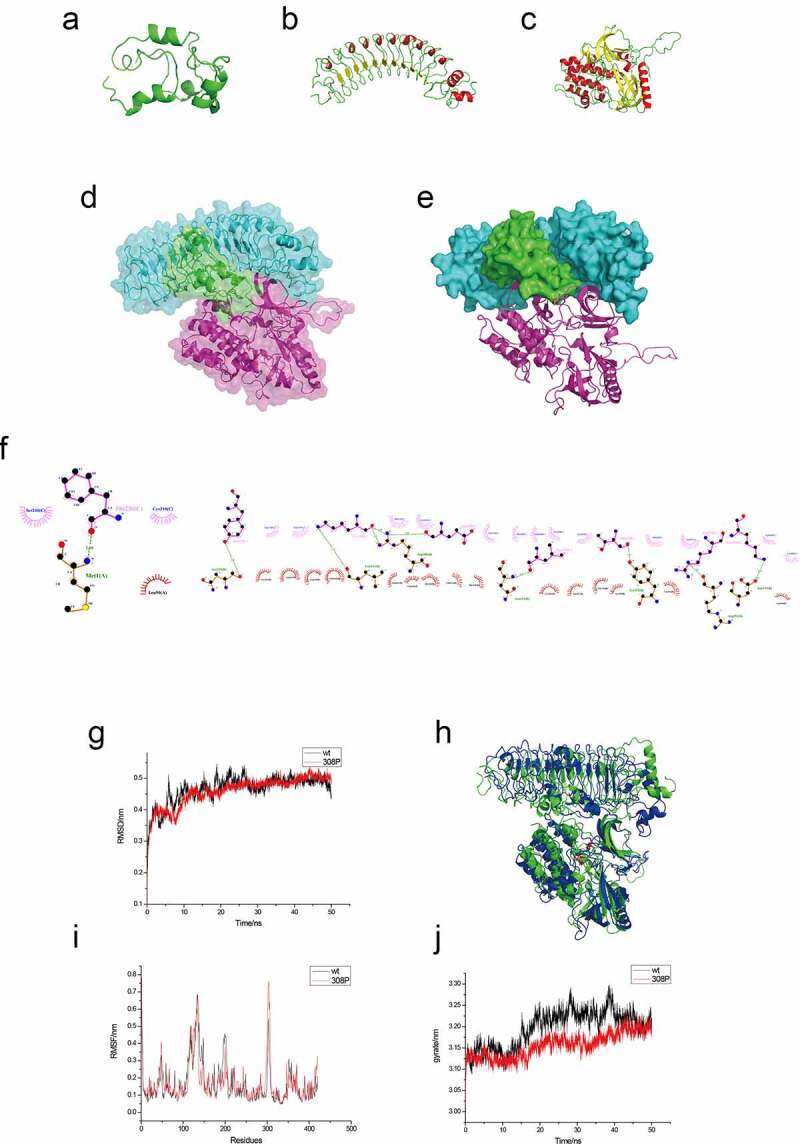
Protein modeling, molecular docking and molecular dynamics simulation. The 3D protein ribbon structure models of (a) MLLT11, (b) TRIL and (c) AKT. (d-e) The 3D surface structure model schematic diagrams of docking between MLLT11(green)-TRIL(cyan) and AKT (violet). (f) The 2D Ligplot represented the interaction site and force between MLLT11-TRIL and AKT. (g) RMSD comparison of wild-type and phosphorylated protein. (h) Complex structure after 50 ns (green for wild-type and blue for phosphorylated protein). (i) Comparison of wild-type and phosphorylated protein RMSF in molecular dynamics process. (j) Comparison of wild-type and phosphorylated protein gyration radius.

We used in silico protein analyses to study the interaction of the proteins. Our protein modeling results showed that the MLLT11 protein was mainly composed of α helixes and loops, while the overall structure was a globular protein (Figure 7(a)). The TRIL protein structure was an arc-shaped rod-like protein, which was mainly composed of 11 β sheets, loops, and short helixes (Figure 7(b)). AKT protein is a globular protein, mainly composed of 13 β sheets, 14 α helixes, and loops (Figure 7(c)). Evaluation by Procheck Server showed that MLLT11, TRIL, and AKT amino acid residues account for more than 95% within a reasonable range, and their structures favored molecular simulation experiments (supplementary Figure S2).

Protein docking showed high affinity between MLLT11-TRIL and AKT. We chose the lowest energy conformation and simulated the three-dimensional (3D) structure of the protein complex (Figure 7(d,e)). The main interaction forces between the AKT protein and MLLT11/TRIL complex were hydrophobic, van der Waals, hydrogen bonding, and electrostatic forces. The AKT protein PHE236 (C) forms a hydrogen bond with the N-terminal MET1 residue of MLLT11 (A); GLU149, LEU153, LYS158, LYS168, TYR175, THR219. In addition, the ARG222 residues of AKT (C) protein form hydrogen bonds with ARG288, ASN311, ASP355, ARG288, ASN239, TYR352, and ARG353 of TRIL (B) protein, respectively (Figure 7(f)). These hydrogen bonds increased the interaction between the three proteins.

Through 50 ns dynamics analysis, the wild-type AKT and p-AKTThr308 protein complexes were almost approaching equilibrium after 30 ns. Thus, the protein conformation gradually stabilized after 30 ns (Figure 7(g)). In addition, between 0 and 30 ns, the root mean square deviation (RMSD) fluctuation of the wild-type protein complex was greater than that of the phosphorylated type, indicating that the MLLT11/TRIL/p-AKTThr308 complex was more stable compared to the MLLT11/TRIL/AKT complex during molecular dynamics.

We then extracted the conformation of 50 ns molecular dynamics as the model analysis and showed that the compactness of the structure of protein complex was enhanced after phosphorylation, which was conducive for the stability of the three proteins (Figure 7(h)).

In addition, a comparative analysis of root mean square fluctuation (RMSF) results indicated that 100–150 residues and 300 residues in the AKT protein modeling structure fluctuated more than other regions. Thus, we speculated that the amino acids in this region defined the functions of the protein (Figure 7(i)). Compared with the wild-type AKT, the 300 amino acid region of the phosphorylated protein had significant fluctuation. We speculated that the structural changes of Thr308 amino acids in this region would enhance the stability of the protein complex.

Furthermore, compared with the wild-type AKT, the phosphorylated AKT had a smaller gyration radius and a small fluctuation range in the molecular dynamics process (Figure 7(j)). The difference in the radius of gyration represents the difference in the stability of the protein complex. The data showed that phosphorylation could stabilize the protein complex.

Through the analysis of the binding free energy of the protein complex, we showed that the total binding free energy of the wild-type protein complex was stable at about −743.6915 kJ/mol, and the total binding free energy of the phosphorylated protein complex was stable at −793.515 kJ/mol (supplementary Table S2). Thus, the interaction of the phosphorylated AKT protein and the MLLT11/TRIL protein was more stable.

**Figure 8.**
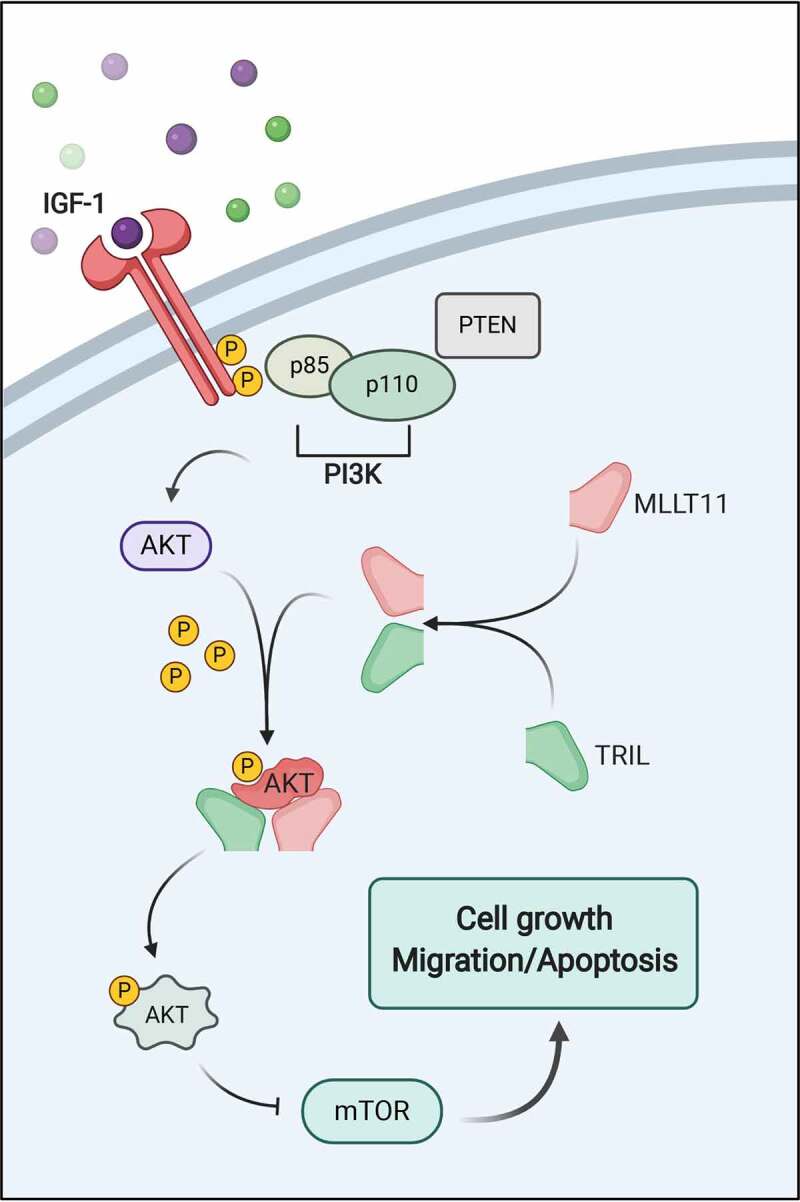
The schematic diagram of MLLT11-TRIL’s involvement in PI3K/AKT/mTOR signaling pathway.

Together, we described, through a schematic diagram, the involvement of MLLT11-TRIL in PI3K/AKT/mTOR signaling pathway (Figure 8). Briefly, MLLT11 forms a complex with TRIL, which then associated with AKT to form a heteromultimer. The latter changes the molecular conformation of AKT, leading to easy phosphorylation of its threonine at position 308, which is more conducive to the transmission of downstream signals.

## 4. Discussion

The PI3K/AKT/mTOR signaling pathway has been extensively studied and has been shown to regulate several biological activities in cells. The processes include cell proliferation, apoptosis, invasion, migration, and autophagy.^26^ Previous data have shown that the pathway is abnormally activated in 80% of ECs.^27^ In addition, available data has shown that inhibition of PI3K/AKT/mTOR signaling pathway is beneficial in the treatment of EC.^28–30^

Insulin-like growth factor 1 (IGF-1) is a cytokine that promotes cell proliferation and resistance to apoptosis. Studies have shown that the IGF-1 mediates many biological processes, and it is closely associated with the occurrence and development of EC in cells.^31–33^ Besides, IGF-1 enhances the proliferation and metastasis of various cancers.^34–38^ In addition, IGF-1 has been shown to exert its biological effects through activation of the PI3K/AKT signaling pathway.^39–41^ This study evaluated the role of IGF-1, an agonist of the PI3K/AKT/mTOR signaling pathway, and showed that IGF-1 could effectively promote the phosphorylation of molecules in the PI3K/AKT/mTOR pathway.

As the central molecule of the PI3K/AKT/mTOR pathway, AKT activates and regulates multiple downstream targets. It promotes protein synthesis and cell growth by activating mTOR and regulate cell proliferation by inactivating cell cycle inhibitors.^42^ In addition, AKT is activated by phosphorylation which then promotes cell cycle progression by regulating glycogen synthesis kinase 3β (GSK3β) and cyclin D1.^43,44^ In vitro, it was shown that Sunitinib could inhibit AKT and sensitize EC cells to ionizing radiation.^45^ Here, we employed MK2206, an inhibitor of AKT, which has been shown to enhance sensitivity to chemotherapy and antitumor activity in EC.^46^ In agreement, we showed that the MK2206 exerts its antitumor activity by inhibiting AKT. However, how the AKT protein is modified and the specific regulation mechanisms remain unknown.

MLLT11 is highly expressed in a variety of tumors, and acts as an oncogene in the activation of a variety of signaling pathways leading to tumor progression.^47^ MLLT11 participates in the WNT and STAT signaling and mediates the occurrence of tumors.^14,48^ Jingwei Hu et al. showed that MLLT11 could regulate the progression of colorectal cancer through AKT.^15^ On the other hand, as a component of the TLR4 signaling complex, TRIL plays a vital role in the inflammatory response.^17,49^ To date, data on the roles of TRIL in tumor development and the specific molecular mechanisms of MLLT11 in regulating AKT remain scant.

Surprisingly, our data show an interaction between MLLT11 and TRIL. This is a previously unreported protein interaction mode. Although MLLT11 and TRIL are known genes, and their functions have also been reported to some extent. Our knowledge of many known molecules is still far from enough. Interactions between different molecules may inspire additional functions, which we hope to explore.

In this study, we found that MLLT11-TRIL can bind to AKT and facilitate the phosphorylation of AKT, thereby indirectly activating the PI3K/AKT downstream signaling pathway. These data suggest that there may be a complex molecular regulatory mechanism for the modification of AKT protein. Digging deep into the molecular role is conducive to the precise development of targeted therapy solutions.

## 5. Conclusions

Taken together, our data show that the MLLT11-TRIL protein complex acts as an AKT agonist and stimulates the activity of the PI3K/AKT/mTOR signaling pathway. This complex binds to and promotes the phosphorylation of AKT protein and may be a potential target for the treatment of EC.

## Supplementary Material

Supplemental Material

## Figures and Tables

**Table:** 

EC	Endometrial cancer
AKT/PKB	Protein kinase B
PI3K	Phosphatidylinositide 3-kinases
PDK1	3-phosphoinositide-dependent protein kinase-1
IGF-1	Insulin-like growth factor 1
MLLT11	MLLT11 transcription factor 7 cofactor
mTOR	Mechanistic target of rapamycin kinase
TRIL	TLR4 interactor with leucine rich repeats
UCEC	Uterine corpus endometrial carcinoma
TCGA	The Cancer Genome Atlas Project
MERAV	Metabolic gEne RApid Visualizer
WGCNA	Weighted gene co-expression network analysis
qRT-PCR	Quantitative real-time PCR
MTT	3-(4,5-dimethyl-2-thiazyl)-2, 5-diphenyl-2H-tetrazolium bromide
SDS-PAGE	Sodium dodecyl sulfate-polyacrylamide gel electrophoresis
PVDF	Polyvinylidene difluoride
IHC	Immunohistochemistry
IF	Immunofluorescence
Co-IP	Co-immunoprecipitation
MD	Molecular dynamics
PME	Particle mesh ewald
RMSD	Root mean square deviation
RMSF	Root mean square fluctuation

## Data Availability

The data that support the findings of this study are openly available in the MERAV (MERAV, http://merav.wi.mit.edu) database and the TCGA data portal (https://tcga-data.nci.nih.gov/tcga/). All other data are available from the authors upon reasonable request.

## References

[1] Eritja N, Chen BJ, Rodriguez-Barrueco R, Santacana M, Gatius S, Vidal A, Martí MD, Ponce J, Bergadà L, Yeramian A, et al. Autophagy orchestrates adaptive responses to targeted therapy in endometrial cancer. Autophagy. 2017;13(3):608–624. doi:10.1080/15548627.2016.1271512. PMID:28055301. 28055301 PMC5361596

[2] Siegel RL, Miller KD, Jemal A. Cancer statistics, 2018. CA Cancer J Clin. 2018;68(1):7–30. doi:10.3322/caac.21442. PMID:29313949. 29313949

[3] Leskela S, Perez-Mies B, Rosa-Rosa JM, Cristobal E, Biscuola M, Palacios-Berraquero ML. Molecular basis of tumor heterogeneity in endometrial carcinosarcoma. Cancers (Basel). 2019;11(7):964. doi:10.3390/cancers11070964. PMID:31324031. PMC6678708 31324031

[4] Yeramian A, Moreno-Bueno G, Dolcet X, Catasus L, Abal M, Colas E, Reventos J, Palacios J, Prat J, Matias-Guiu X, et al. Endometrial carcinoma: molecular alterations involved in tumor development and progression. Oncogene. 2013;32(4):403–413. doi:10.1038/onc.2012.76. PMID:22430211. 22430211

[5] Song J, Chen W, Zhu G, Wang W, Sun F, Zhu J. Immunogenomic profiling and classification of prostate cancer based on HIF-1 signaling pathway. Front Oncol. 2020;10:1374. doi:10.3389/fonc.2020.01374. PMID:32850440. 32850440 PMC7425731

[6] Bokhman JV. Two pathogenetic types of endometrial carcinoma. Gynecol Oncol. 1983;15(1):10–17. doi:10.1016/0090-8258(83)90111-7. PMID:6822361. 6822361

[7] Elmenier FM, Lasheen DS, Abouzid K. Phosphatidylinositol 3 kinase (PI3K) inhibitors as new weapon to combat cancer. Eur J Med Chem. 2019;183:111718. doi:10.1016/j.ejmech.2019.111718. PMID:31581005. 31581005

[8] Hers I, Vincent EE, Tavare JM. Akt signalling in health and disease. Cell Signal. 2011;23(10):1515–1527. doi:10.1016/j.cellsig.2011.05.004. PMID:21620960. 21620960

[9] Heron-Milhavet L, Khouya N, Fernandez A, Lamb NJ. Akt1 and Akt2: differentiating the aktion. Histol Histopathol. 2011;26(5):651–662. doi:10.14670/HH-26.651. PMID:21432781. 21432781

[10] Ding Z, Liang J, Li J, Lu Y, Ariyaratna V, Lu Z, Davies MA, Westwick JK, and Mills GB . Physical association of PDK1 with AKT1 is sufficient for pathway activation independent of membrane localization and phosphatidylinositol 3 kinase. Plos One. 2010;5(3):e9910. doi:10.1371/journal.pone.0009910. PMID:20361045. 20361045 PMC2845649

[11] Vejux A, Guyot S, Montange T, Riedinger JM, Kahn E, Lizard G. Phospholipidosis and down-regulation of the PI3-K/PDK-1/Akt signalling pathway are vitamin E inhibitable events associated with 7-ketocholesterol-induced apoptosis. J Nutr Biochem. 2009;20(1):45–61. doi:10.1016/j.jnutbio.2007.12.001. PMID:18495460. 18495460

[12] Di Maira G, Brustolon F, Pinna LA, Ruzzene M. Dephosphorylation and inactivation of Akt/PKB is counteracted by protein kinase CK2 in HEK 293T cells. Cell Mol Life Sci. 2009;66(20):3363–3373. doi:10.1007/s00018-009-0108-1. PMID:19662498. 19662498 PMC11115639

[13] Tse W, Zhu W, Chen HS, Cohen A. A novel gene, AF1q, fused to MLL in t(1;11) (q21;q23), is specifically expressed in leukemic and immature hematopoietic cells. Blood. 1995;85(3):650–656. doi:10.1182/blood.V85.3.650.bloodjournal853650. PMID:7833468. 7833468

[14] Park J, Schlederer M, Schreiber M, Ice R, Merkel O, Bilban M, Hofbauer S, Kim S, Addison J, Zou J, et al. AF1q is a novel TCF7 co-factor which activates CD44 and promotes breast cancer metastasis. Oncotarget. 2015;6(24):20697–20710. doi:10.18632/oncotarget.4136. PMID:26079538. 26079538 PMC4653036

[15] Hu J, Li G, Liu L, Wang Y, Li X, Gong J. AF1q mediates tumor progression in colorectal cancer by regulating AKT signaling. Int J Mol Sci. 2017;18(5):987. doi:10.3390/ijms18050987. PMID:28475127. PMC5454900 28475127

[16] Man G, Duan A, Liu W, Cheng J, Liu Y, Song J, Zhou H, Shen K. Circular RNA-related CeRNA network and prognostic signature for patients with osteosarcoma. Cancer Manag Res. 2021;13:7527–7541. doi:10.2147/CMAR.S328559. PMID:34629900. 34629900 PMC8494289

[17] Carpenter S, Carlson T, Dellacasagrande J, Garcia A, Gibbons S, Hertzog P, Lyons A, Lin L-L, Lynch M, Monie T, et al. TRIL, a functional component of the TLR4 signaling complex, highly expressed in brain. J Immunol. 2009;183(6):3989–3995. doi:10.4049/jimmunol.0901518. PMID:19710467. 19710467

[18] Wang M, Liao J, Wang J, Qi M, Wang K, Wu W. TAF1A and ZBTB41 serve as novel key genes in cervical cancer identified by integrated approaches. Cancer Gene Ther. 2021;28(12):1298–1311. doi:10.1038/s41417-020-00278-1. PMID:33311601. 33311601 PMC8636252

[19] Shaul YD, Yuan B, Thiru P, Nutter-Upham A, McCallum S, Lanzkron C, Bell GW, Sabatini DM. MERAV: a tool for comparing gene expression across human tissues and cell types. Nucleic Acids Res. 2016;44(D1):D560–D566. doi:10.1093/nar/gkv1337. PMID:26626150. 26626150 PMC4702927

[20] Tomczak K, Czerwinska P, Wiznerowicz M. The Cancer Genome Atlas (TCGA): an immeasurable source of knowledge. Contemp Oncol (Pozn). 2015;19(1A):A68–A77. doi:10.5114/wo.2014.47136. PMID:25691825. 25691825 PMC4322527

[21] Wang M, Wang J, Liu J, Zhu L, Ma H, Zou J, Wu W, Wang K. Systematic prediction of key genes for ovarian cancer by co-expression network analysis. J Cell Mol Med. 2020;24(11):6298–6307. doi:10.1111/jcmm.15271. PMID:32319226. 32319226 PMC7294139

[22] Raudvere U, Kolberg L, Kuzmin I, Arak T, Adler P, Peterson H, Vilo J. g:Profiler: a web server for functional enrichment analysis and conversions of gene lists (2019 update). Nucleic Acids Res. 2019;47(W1):W191–W198. doi:10.1093/nar/gkz369. PMID:31066453. 31066453 PMC6602461

[23] Wang M, Liao J, Tan C, Zhou H, Wang J, Wang K, Li Y, Wu W. Integrated study of miR-215 promoting breast cancer cell apoptosis by targeting RAD54B. J Cell Mol Med. 2021;25(7):3327–3338. doi:10.1111/jcmm.16402. PMID:33635591. 33635591 PMC8034472

[24] Liao J, Liu J, Wang J, Wang M. Lnc-CPLC promotes the progression of colorectal cancer via regulating ZBTB34 by competitively binding miR-4319. J Cell Physiol. 2021. doi:10.1002/jcp.30628. PMID:34741317. 34741317

[25] Kim DE, Chivian D, Baker D. Protein structure prediction and analysis using the Robetta server. Nucleic Acids Res. 2004;32(Web Server issue):W526–W531. doi:10.1093/nar/gkh468. PMID:15215442. 15215442 PMC441606

[26] Aoki M, Fujishita T. Oncogenic Roles of the PI3K/AKT/mTOR Axis. Curr Top Microbiol Immunol. 2017;407:153–189. doi:10.1007/82_2017_6. PMID:28550454. 28550454

[27] Sorolla MA, Parisi E, Sorolla A. Determinants of sensitivity to radiotherapy in endometrial cancer. Cancers (Basel). 2020;12(7):1906. doi:10.3390/cancers12071906. PMID:32679719. PMC7409033 32679719

[28] Miyasaka A, Oda K, Ikeda Y, Sone K, Fukuda T, Inaba K, Makii C, Enomoto A, Hosoya N, Tanikawa M, et al. PI3K/mTOR pathway inhibition overcomes radioresistance via suppression of the HIF1-alpha/VEGF pathway in endometrial cancer. Gynecol Oncol. 2015;138(1):174–180. doi:10.1016/j.ygyno.2015.04.015. PMID:25913131. 25913131

[29] Roncolato F, Lindemann K, Willson ML, Martyn J, Mileshkin L. PI3K/AKT/mTOR inhibitors for advanced or recurrent endometrial cancer. Cochrane Database Syst Rev. 2019;10:D12160. doi:10.1002/14651858.CD012160.pub2. PMID:31588998. PMC6953296 31588998

[30] Barra F, Evangelisti G, Ferro DL, Di Domenico S, Ferraioli D, Vellone VG, De Cian F, Ferrero S. Investigational PI3K/AKT/mTOR inhibitors in development for endometrial cancer. Expert Opin Investig Drugs. 2019;28(2):131–142. doi:10.1080/13543784.2018.1558202. PMID:30574817. 30574817

[31] Majchrzak-Baczmanska D, Malinowski A. Does IGF-1 play a role in the biology of endometrial cancer? Ginekol Pol. 2016;87(8):598–604. doi:10.5603/GP.2016.0052. PMID:27629137. 27629137

[32] Kwasniewski W, Gozdzicka-Jozefiak A, Wolun-Cholewa M, Polak G, Sierocinska-Sawa J, Kwasniewska A, Kotarski J. Microsatellite polymorphism in the P1 promoter region of the IGF-1 gene is associated with endometrial cancer. Mol Med Rep. 2016;13(6):4950–4958. doi:10.3892/mmr.2016.5181. PMID:27121258. 27121258 PMC4878573

[33] Zhang Y, Li MX, Wang H, Zeng Z, Li XM. Metformin down-regulates endometrial carcinoma cell secretion of IGF-1 and expression of IGF-1R. Asian Pac J Cancer Prev. 2015;16(1):221–225. doi:10.7314/apjcp.2015.16.1.221. PMID:25640355. 25640355

[34] Anisimov VN, Bartke A. The key role of growth hormone–insulin–IGF-1 signaling in aging and cancer. Crit Rev Oncol Hematol. 2013;87(3):201–223. doi:10.1016/j.critrevonc.2013.01.005. PMID:23434537. 23434537 PMC4095988

[35] Majchrzak-Baczmanska D, Malinowski A, Glowacka E, Wilczynski M. Does IGF-1 play a role in the biology of ovarian cancer? Ginekol Pol. 2018;89(1):13–19. doi:10.5603/GP.a2018.0003. PMID:29411341. 29411341

[36] Min DY, Jung E, Kim J, Lee YH, Shin SY. Leptin stimulates IGF-1 transcription by activating AP-1 in human breast cancer cells. BMB REP. 2019;52(6):385–390. doi:10.5483/BMBRep.2019.52.6.189. PMID:30293548. 30293548 PMC6605521

[37] Roberts CJ. IGF-1 and prostate cancer. Novartis Found Symp. 2004;262:193–199, 199–204, 265–268. PMID:15562830. 15562830

[38] Lonning PE, Helle SI. IGF-1 and breast cancer. Novartis Found Symp. 2004;262:205–212, 212–214, 265–268. PMID:15562831. 15562831

[39] Yang L, Wang H, Liu L, Xie A. The role of Insulin/IGF-1/PI3K/Akt/GSK3beta signaling in Parkinson’s disease dementia. Front Neurosci. 2018;12:73. doi:10.3389/fnins.2018.00073. PMID:29515352. 29515352 PMC5826217

[40] Timmer LT, Hoogaars W, Jaspers RT. The role of IGF-1 signaling in skeletal muscle atrophy. Adv Exp Med Biol. 2018;1088:109–137. doi:10.1007/978-981-13-1435-3_6. PMID:30390250. 30390250

[41] Rong L, Li Z, Leng X, Li H, Ma Y, Chen Y, Song F. Salidroside induces apoptosis and protective autophagy in human gastric cancer AGS cells through the PI3K/Akt/mTOR pathway. Biomed Pharmacother. 2020;122:109726. doi:10.1016/j.biopha.2019.109726. PMID:31918283. 31918283

[42] Ersahin T, Tuncbag N, Cetin-Atalay R. The PI3K/AKT/mTOR interactive pathway. Mol Biosyst. 2015;11(7):1946–1954. doi:10.1039/c5mb00101c. PMID:25924008. 25924008

[43] Yuan Y, Fan Y, Gao Z, Sun X, Zhang H, Wang Z, Cui Y, Song W, Wang Z, Zhang F, et al. SHP2 promotes proliferation of breast cancer cells through regulating Cyclin D1 stability via the PI3K/AKT/GSK3beta signaling pathway. Cancer Biol Med. 2020;17(3):707–725. doi:10.20892/j.2095-3941.2020.0056. PMID:32944401. 32944401 PMC7476086

[44] Xu S, Zhang H, Liu T, Yang W, Lv W, He D, Guo P, Li L. 6-Gingerol induces cell-cycle G1-phase arrest through AKT-GSK 3beta-cyclin D1 pathway in renal-cell carcinoma. Cancer Chemother Pharmacol. 2020;85(2):379–390. doi:10.1007/s00280-019-03999-9. PMID:31832810. 31832810 PMC7015962

[45] Wang E, Sorolla A. Sensitizing endometrial cancer to ionizing radiation by multi-tyrosine kinase inhibition. J Gynecol Oncol. 2020;31(3):e29. doi:10.3802/jgo.2020.31.e29. PMID:31912683. 31912683 PMC7189072

[46] Hirai H, Sootome H, Nakatsuru Y, Miyama K, Taguchi S, Tsujioka K, Ueno Y, Hatch H, Majumder PK, Pan B-S, et al. MK-2206, an allosteric akt inhibitor, enhances antitumor efficacy by standard chemotherapeutic agents or molecular targeted drugs in vitro and in vivo. Mol Cancer Ther. 2010;9(7):1956–1967. doi:10.1158/1535-7163.MCT-09-1012. PMID:20571069. 20571069

[47] Khan N, Park J, Dean WL, Gray RD, Tse W, Lee D, Sabo TM. Recombinant expression and purification of AF1q and its interaction with T-cell Factor 7. Protein Expr Purif. 2020;165:105499. doi:10.1016/j.pep.2019.105499. PMID:31541685. 31541685

[48] Gruber ES, Oberhuber G, Birner P, Schlederer M, Kenn M, Schreiner W, Jomrich G, Schoppmann SF, Gnant M, and Tse W. The oncogene AF1Q is associated with WNT and STAT signaling and offers a novel independent prognostic marker in patients with resectable esophageal cancer. Cells-Basel. 2019;8(11). doi:10.3390/cells8111357. PMID:31671695. PMC6912824 31671695

[49] Jia H, Ma H, Li Z, Chen F, Fang B, Cao X, Chang Y, Qiang Z. Downregulation of LncRNA TUG1 inhibited TLR4 signaling pathway-mediated inflammatory damage after spinal cord ischemia reperfusion in rats via suppressing TRIL expression. J Neuropathol ExpNeurol.2019;78(3):268–282.doi:10.1093/jnen/nly126. PMID:30715406. 30715406

