# Supplementary material for: MLLT11-TRIL complex promotes the progression of endometrial cancer through PI3K/AKT/mTOR signaling pathway
Source: Cancer Biol Ther. 2022 Mar 6;23(1):211–24. doi: 10.1080/15384047.2022.2046450 (PMC8903758; doi:10.1080/15384047.2022.2046450)
Supplement: Supplemental Material [file KCBT_A_2046450_SM6885.pdf]

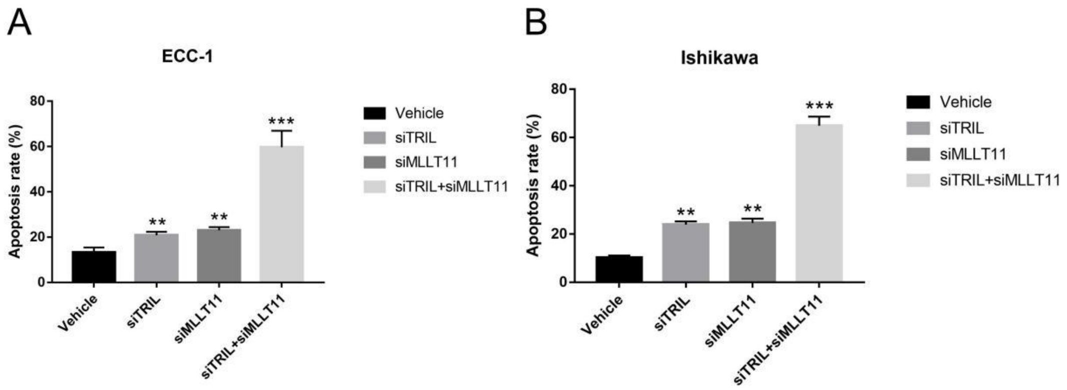

Supplementary figure 1

After knocking down TRIL, MLLT11 and MLLT11-TRIL were co-knockdown with siRNA, histograms of the apoptosis rate of the two cell lines (A) ECC-1 and (B) Ishikawa.

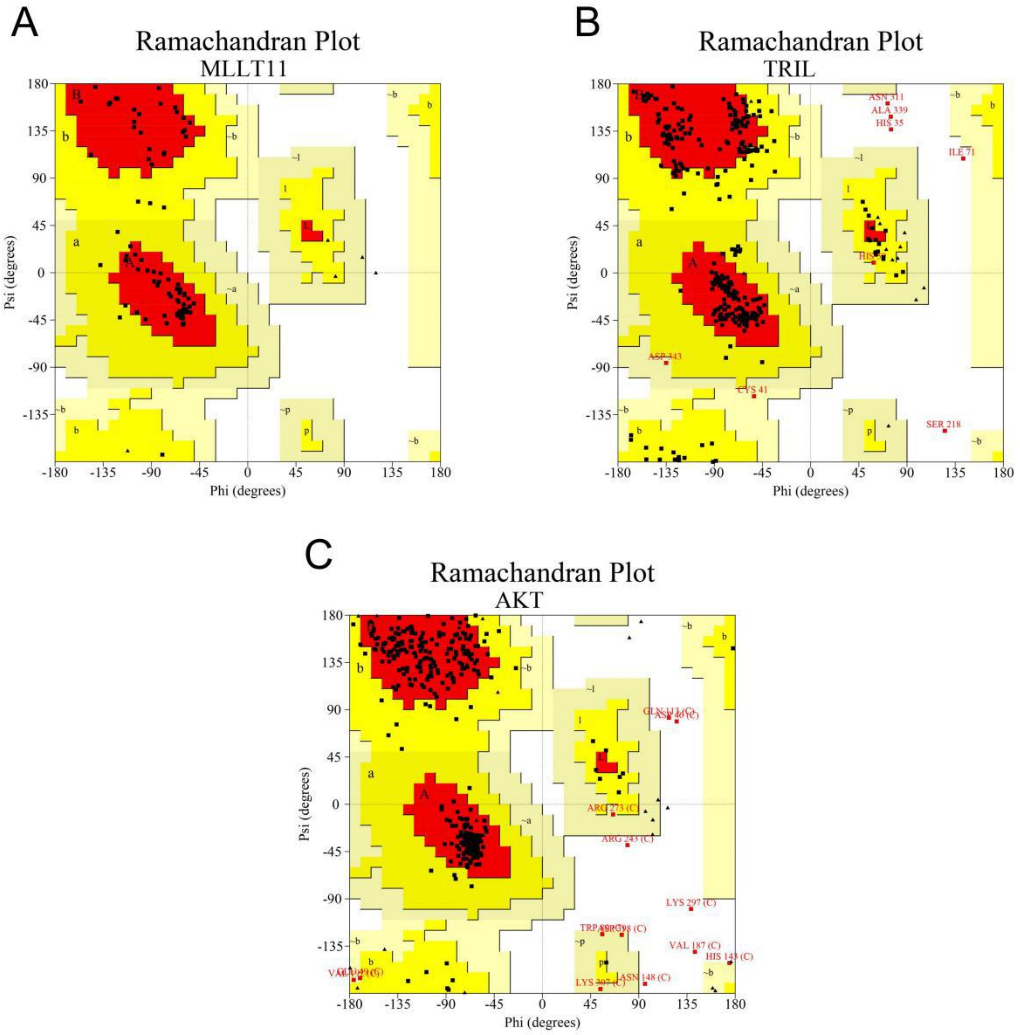

Supplementary figure 2

Ramachandran plots of MLLT11, TRIL and AKT proteins. Red is the best area; Yellow is the general area; White is not an allowed area.

Supplementary table 1: Amino acid sequences modeled for MLLT11, TRIL and AKT1.

**The amino acid sequence of MLLT11:**
MRDPVSSQYSSFLFWRMPIPELDLSELEGLGLSDTATYKVKDSSVGKMIGQAT<br>AADQEKNPEGDGLLEYSTFNFWRAPIASIHSFELDLL

**The amino acid sequence of TRIL:**
EAARALRLLLVVCGCLALPPLAEPVCPERCDCQHPQHLLCTNRGLRVVPKTSS<br>LPSPHDVLTYSLGGNFITNITAFDFHRLGQLRRLDLQYNQIRSLHPKTFEKLSRL<br>EELYLGNNLLQALAPGTLAPLRKLRILYANGNEISRLSRGSFEGLESLVKLRLD<br>GNALGALPDAVFAPLGNLLYLHLESNRIRFL

**The amino acid sequence of AKT1:**
MSDVAIVKEGWLHKRGEYIKTWRPRYFLLKNDGTFIGYKERPQDVDQREAPL<br>NNFSVAQCQLMKTERPRPNTFIIRCLQWTTVIERTFHVETPEEREEWTTAIQTV<br>ADGLKKQEEEEMDFRSGSPSDNSGAEEMEVSLAKPKHRVTMNEFEYLKLLG<br>KGTFGKVILVKEKATGRYYAMKILKKEVIVAKDEVAHTLTENRVLQNSRHPFL<br>TALKYSFQTHDRLCFVMEYANGGELFFHLSRERVFSEDRARFYGAEIVSALDY<br>LHSEKNVVYRDLKLENLMLDKDGHIKITDFGLCKEGIKDGATMKTFCGTPEY<br>LAPEVLEDNDYGRAVDWWGLGVVMYEMMCGRLPFYNQDHEKLFELILMEE<br>IRFPRTLGPEAKSLLSGLLKKDPKQRLGGGSEDAKEIMQHRFFAGIVWQHVYE<br>KKLSPPFKPQVTSETDTRYFDEEFTAQMITITPPDQDDSMECVDSERRPHFPQF<br>SYSASGTA

Supplementary table 2: Analysis of binding free energy of wild-type protein complex and phosphorylated protein complex

Interaction type	Akt protein complex binding free energy（kJ/mol)	p-Akt protein complex binding free energy（kJ/mol)
van der Waal energy	-417.345±126.423	-571.739±65.903
Electrostattic energy	-2217.650±256.618	-1828.090±162.350
Polar solvation energy	1950.939±323.629	1678.957±290.908
SASA energy	-59.635±14.000	-72.643±5.733
Binding energy	-743.691±181.082	-793.515±224.123

Supplementary table 3：The clinicopathologic characteristics of the 50 patients

Patient number	Age at diagnosis	Histologic grade	Clinical stage
1	55	G3	Stage IB
2	49	G3	Stage IB
3	87	G3	Stage IB
4	60	G3	Stage IVA
5	54	G3	Stage IB
6	51	G3	Stage IIIC
7	71	G1	Stage I
8	52	G2	Stage IIIA
9	34	G3	Stage IA
10	62	G2	Stage II
11	87	G2	Stage II
12	61	G2	Stage IA
13	73	G3	Stage II
14	60	G3	Stage IIIC1
15	70	G3	Stage IA
16	55	G3	Stage IA
17	86	G2	Stage I
18	85	G1	Stage IIIA
19	64	G2	Stage IIIB
20	73	G3	Stage IIIA
21	77	G1	Stage IB
22	64	G3	Stage IB
23	78	G3	Stage IB
24	53	G2	Stage IA
25	65	G3	Stage IB
26	56	G3	Stage IVB
27	64	G3	Stage IA
28	58	G2	Stage IA
29	42	G1	Stage II
30	75	G1	Stage IA
31	61	G3	Stage IA
32	56	G1	Stage IB
33	83	G3	Stage IB
34	78	G3	Stage IIIC1
35	67	G1	Stage IA
36	72	G3	Stage IA
37	60	G3	Stage IIIC2
38	55	G3	Stage IB

39	72	G3	Stage IVB
40	68	G1	Stage IA
41	74	G3	Stage II
42	57	G3	Stage IA
43	61	G2	Stage IA
44	61	G2	Stage II
45	59	G2	Stage IIIB
46	69	G2	Stage IB
47	76	G2	Stage III
48	41	G2	Stage IA
49	64	G3	Stage IA
50	74	G3	Stage II
